# An assessment of sensitivity biomarkers for urinary cadmium burden

**DOI:** 10.1186/s12882-020-02036-9

**Published:** 2020-09-05

**Authors:** Yuting Li, Hongmei Wang, Jie Yu, Qiong Yan, Honggang Hu, Lishu Zhang, Tian Tian, Xianglei Peng, Shuo Yang, Shen Ke

**Affiliations:** 1 Department of Life Science and Bioengineering, School of Science, Beijing Jiaotong University, No.3, Shangyuan Village, Haidian District, Beijing, 100044 China; 2 State Key Laboratory of Environmental Criteria and Risk Assessment, Department of Environment and Health, Chinese Research Academy of Environmental Sciences, Beijing, 100012 PR China

**Keywords:** Urinary cadmium, Urinary N-acetyl-β-D-glucosidase (UNAG), Urinary β_2_-microglobulin (Uβ_2_-MG), Benchmark dose, Renal dysfunction

## Abstract

**Background:**

Excess cadmium (Cd) intake poses a general risk to health and to the kidneys in particular. Among indices of renal dysfunction under Cd burden measures are the urinary N-acetyl-β-D-glucosidase (UNAG) and urinary β_2_-microglobulin (Uβ_2_-MG) enzymes. However, the end-pointed values and the Cd burden threshold remain controversial because the scopes fluctuate widely.

**Methods:**

To ascertain the clinical benchmark dose of urinary Cd (UCd) burden for renal dysfunction, 1595 residents near a Cd site were surveyed. Urine was sampled and assayed. A benchmark dose low (BMDL) was obtained by fitting UCd levels and index levels.

**Results:**

We found that over 50% of the subjects were suffering from Cd exposure as their UCd levels far exceeded the national standard threshold of 5.000 μg/g creatinine (cr). Further analysis indicated that Uβ_2_-MG was more sensitive than UNAG for renal dysfunction. The BMDL for UCd was estimated as 3.486 U/g cr (male, where U is unit of enzyme) and 2.998 U/g cr (female) for UNAG. The BMDL for Uβ_2_-MG, which is released into urine from glomerulus after Cd exposure, was found to be 2.506 μg/g cr (male, where μg is the unit of microglobulin) and 2.236 μg/g cr (female).

**Conclusions:**

Uβ_2_-MG is recommended as the sensitivity index for renal dysfunction, with 2.2 μg/g cr as the threshold for clinical diagnosis. Our findings suggest that Uβ_2_-MG is the better biomarker for exposure to Cd.

## Background

Cadmium (Cd) in the environment enters organisms via the atmosphere, drinking water, and contaminated soil and food [1]. Excess Cd exposure is harmful to health [2, 3]. The well-known case of the Itai-Itai disease in Japan was the most severe stage of long-term Cd exposure [1]. Many organs are damaged by excess Cd burden, resulting in defective bone formation, including osteoporosis [4, 5], and some cancers [6, 7]. The kidney is an important organ that suffers damage from Cd exposure, as reported by previous studies [8, 9]. This view has been further supported by more recent research, which has also recommended that the kidney is to be considered an early-stage target of Cd [10, 11].

A range of factors (including age, years of exposure, and gender) that affect the relationship between Cd burden and the prevalence of kidney dysfunction have been determined to contribute the Cd threshold [12–14], but the interaction mechanism remains unclear. A range of biomarkers of renal dysfunction have been used diagnostically in this context [15, 16], including urinary N-acetyl-β-D-glucosidase (UNAG), urinary β_2_-microglobulin (Uβ_2_-MG), and urinary retinol binding protein (URBP) [17–19]. These three are likely valid biomarkers due to their origin in the renal tubules or in glomeruli breakage under urinary (UCd) pressure [20–22]. However, it is not yet clear which is the most sensitive to UCd.

The threshold concentrations of UCd used in different countries varies due to the differences in subjects and models. The benchmark dose (BMD) is the dose that can increase an effect to a specific response level. The value of BMD can reflect the dose-response relationship and the quality of the data used. The benchmark dose method is a new method to determine the reference dose. This method can explain and develop the dose-response relationship from the perspective of mathematical statistics, and has become an effective method for toxicological science to determine the reference dose. No observed adverse effect level (NOAEL) refers to the highest dose or concentration of an exogenous chemical that does not cause detectable harmful effects in the body through experiments and observations under prescribed exposure conditions. This method ignores the early effects of adverse reactions. When the health threshold of heavy metals is being studied, the benchmark dose (BMD) method can make up for the deficiencies of the No Observed Adverse Effect Level (NOAEL) method. The BMD of UCd obtained from Uβ_2_-MG ranged from 2.08 to 3.80 μg/g creatinine (cr) at one site, and at another, the BMD varied from 0.99 to 3.34 μg/g cr [23]. Another study found a BMD of UCd 1.9 μg/g cr measured by Uβ_2_-MG, from 81 samples [22]. It is speculated that sample size might interfere with UCd threshold levels.

With greater insight into the sensitivity of the biomarkers, the data for the UCd scope was to vary widely, as did the renal dysfunction indexes. For example, the estimated BMD low (BMDL) of UCd was 6.9 μg/g cr (where μg is the unit of microglobulin) for Uβ_2_-MG and 4.4 U/g cr (where U is unit of the enzyme) for UNAG in males and 8.1 μg/g cr for Uβ_2_-MG and 6.1 U/g cr for UNAG in females [24]. In addition, the BMDs for UCd for β_2_-MG and UNAG were 2.09 μg/g cr and 1.80 U/g cr in males and 2.10 μg/g cr and 2.31 U/g cr in females, respectively [25].

To obtain an early diagnosis and prevention for Cd exposure, it is essential to establish the threshold for the sensitivity index for clinical detection. However, there is still little quantitative analysis of the changes in UCd BMDs for the occupationally exposed, who have high Cd exposure levels, in comparison to those who are not occupationally exposed. This study, therefore, assessed the UCd BMDs by sampling people living with high levels of Cd exposure.

## Methods

### Survey location and subjects

Xiaogan in Hubei province, China, was selected as the Cd exposure site for this study [26]. Smelting has been performed here for nearly three decades. The residents of 20 nearby villages could conceivably have been exposed to Cd released directly or indirectly from the smelting. The average age of the participants in this study was more than 50 years, and most had exposure times of more than 25 years. Patients with chronic or endocrine diseases, such as diabetes or thyroid, liver, or kidney damage, were excluded. Some participants had had long-term exposure through their employment.

Participants with missing data, including demographic information, and those with urinary cr lower than 0.3 g/L or above 3.0 g/L, were not factored in. A total of 1595 participants, most of who were engaged in agriculture, were observed in this study. In all, 36.8% (587) were male and 63.2% (1008) were female. All subjects consumed locally produced rice and vegetables and drank municipal water. Questionnaires were administered after that informed consent was signed. This study was approved by the Ethics Committee of the School of Science at Beijing Jiaotong University and of the No. 1 People’s Hospital of Xiaogan. The study was carried out by uniformly trained investigators.

### Sampling and testing

The containers and utensils used for urine collection and transportation were thoroughly washed, soaked with dilute nitric acid for 12 h, and rinsed with deionized water before use. Morning urine samples of the 1595 subjects were collected and then stored frozen at − 20 °C for analysis.

UNAG and Uβ_2_-MG in urine were measured as indexes of renal dysfunction. The UCd concentrations were determined using graphite furnace atomic absorption spectrometry with peak area evaluation described in previous studies. The UNAG concentrations were measured using the spectrophotometer colorimetric method, and the Uβ_2_-MG concentrations were measured using radioimmunoassay. All urine samples were acidified with concentrated nitric acid to determine the different UCd concentrations. Urinary cr concentrations were assessed with picric acid with protein method. All measurement techniques were conducted as described elsewhere [27]. All samples were tested using parallel samples, and the detection value was less than 5%. According to the Guidelines for the Diagnosis and Treatment of Heavy Metal Pollution of China (Trial) in 2010 (GOMOHC) and the Minister of Health of the People’s Republic of China (MOHC) in 1998 [28, 29], the threshold values for UNAG and Uβ_2_-MG are 17 U/g cr and 1000 μg/g cr, respectively. All urinary indexes were adjusted for the cr concentrations, and all were expressed as μg/g cr.

### Statistical analysis

Statistical analysis and hypothesis testing on data were performed by SPSS 22.0. Two independent sample t-tests and the non-parametric Mann–Whitney test were used to compare the means. The UCd levels were transformed by logarithm (log10) to obtain variables that were approximately normally distributed and to meet the linear model requirement before they were detected by Pearson correlation analysis. Abnormalities in health effects were tested using the χ^2^ test. The correlations between UCd burden and exposure factors (including age, years of exposure, and gender) were assessed using a multiple linear regression model (α = 0.05), and *p*-values less than 0.05 were considered to indicate statistically significant differences. Analysis of variance, the χ^2^ test, and Spearman rank correlation were used to detect significant differences in the relationship between the UCd burden and the renal dysfunction index.

### BMD computation

The BMDs of UCd and the 95% lower confidence limits (BMDLs) in relation to renal effects were deduced by Benchmark Dose Software (US Environmental Protection Agency, version 2.7.0.4). The BMDL was set in relation to a health risk assessment. The results were cross-validated using four models (the gamma, logistic, log-logistic, and log-profit models). The Benchmark Dose Technical Guidance asserts that the lower the Akaike information criterion (AIC) value, the better optimized the model derived, and a similar principle holds for χ^2^ values. The small undulation in χ^2^ values is the counterpart to a lower deviation in experimental results, meaning a better-fitting result. Here the lowest AIC value was obtained by logistic model, so it was used to calculate the UCd threshold concentration, on the condition that the benchmark dose response (BMR) was set as an extra 10% risk. Where *p* ≥ 0.05, the results were defined as the endpoint BMD value, following the Benchmark Dose Technical Guidance [30].

## Results

### Demographic information

In all, 1595 (587 male and 1008 female) participants from twenty villages near Xiaogan were surveyed, and the demographic character was shown (Table 1). The average participant age was 63.89 (±8.4) years old (the female average was 65.01 years old and the male average was 63.24). The UCd concentration was in the scope of 0.030 to 20.279 μg/g cr (Table 1). The maximum geometric mean of the male UCd levels (4.726 μg/g cr) was lower than that of females (5.393 μg/g cr). The average UCd of the total population was estimated to be 4.507 μg/g cr. An increasing trend was shown between male and female UCd levels. The geometric mean value for UCd among females was higher than that for males (*p* < 0.05). The mean UNAG value among the males (11.790 U/g cr) was different from that for females (11.817 U/g cr). The average male Uβ_2_-MG level was 611.63 μg/g cr, and the female average was 643.10 μg/g cr (Table 1). The mean UNAG (11.807 U/g cr) for all subjects was lower than the national standard threshold value (17 U/g cr) (*p* < 0.05) as found by MOHC in 1998, and the Uβ_2_-MG mean (631.33 μg/g cr) was lower than 1000 μg/g cr (*p* < 0.05). Further analysis revealed that the maximum value found for UNAG was 3.89 times the national standard threshold value, and the Uβ_2_-MG value was 2.97 times the standard. There was a large undulation shown in the scope of UNAG, which varied from 0.411 to 66.157 U/g cr, and Uβ_2_-MG changed from 4.09 to 2965.66 μg/g cr.

**Table 1:** Characteristics of the population entailed in the study

Gender	Age	No.	Age	UCd	Range	UNAG	Range	Uβ_2_-MG	Range
Male	[50,60]	188	56.00 (2.984)	4.387 (3.122)	0.046 ~ 18.397	12.193 (9.176)	1.160 ~ 46.516	634.34 (527.703)	7.83 ~ 2497.43
	(60,70]	263	65.24 (2.655)	4.493 (3.878)	0.044 ~ 17.859	11.353 (9.414)	0.631 ~ 40.485	594.95 (524.558)	4.30 ~ 2518.24
	(70,80]	104	74.88 (2.720)	4.560 (4.075)	0.046 ~ 18.397	11.010 (9.147)	0.838 ~ 43.640	615.05 (568.154)	10.30 ~ 2573.07
	(80,90]	32	84.06 (2.449)	4.726 (5.105)	0.500 ~ 18.662	16.493 (11.883)	3.899 ~ 66.157	608.48 (536.842)	75.79 ~ 2943.91
	Total	587	65.01 (8.408)	4.483 (3.786)	0.044 ~ 18.925	11.790 (9.527)	0.631 ~ 66.157	611.63 (535.198)	4.30 ~ 2943.91
Female	[50,60]	401	55.20 (3.297)	4.408 (3.878)	0.085 ~ 20.279	11.581 (8.794)	0.435 ~ 41.642	615.36 (492.337)	4.09 ~ 2552.80
	(60,70]	424	65.06 (2.625)	4.494 (3.804)	0.030 ~ 18.905	12.001 (9.698)	0.505 ~ 46.664	652.13 (575.076)	12.37 ~ 2614.23
	(70,80]	153	75.17 (2.837)	4.748 (4.162)	0.320 ~ 19.694	12.240 (10.138)	0.921 ~ 47.250	678.00 (604.090)	37.78 ~ 2828.72
	(80,90]	30	84.13 (2.872)	5.393 (3.889)	0.884 ~ 16.330	10.400 (10.563)	0.411 ~ 50.105	727.37 (648.506)	42.09 ~ 2965.66
	Total	1008	63.24 (8.392)	4.522 (3.896)	0.030 ~ 20.279	11.817 (9.462)	0.411 ~ 50.105	643.10 (553.120)	4.09 ~ 2965.66
Total	[50,60]	589	55.45 (3.222)	4.401 (3.657)	0.046 ~ 20.279	11.773 (8.923)	0.435 ~ 46.516	621.35 (504.178)	4.09 ~ 2552.80
	(60,70]	687	65.13 (2.638)	4.494 (3.833)	0.030 ~ 18.905	11.749 (9.595)	0.505 ~ 46.664	629.62 (557.553)	4.30 ~ 2614.23
	(70,80]	257	75.05 (2.794)	4.671 (4.127)	0.095 ~ 19.694	11.726 (9.773)	0.838 ~ 47.250	651.78 (589.980)	10.30 ~ 2828.72
	(80,90]	62	84.10 (2.662)	5.038 (4.558)	0.500 ~ 18.662	13.194 (11.527)	0.411 ~ 66.157	663.36 (600.832)	42.09 ~ 2965.66
	Total	1595	63.89 (8.442)	4.507 (3.856)	0.030 ~ 20.279	11.807 (9.486)	0.411 ~ 66.157	631.33 (546.768)	4.09 ~ 2965.66

The units of the above indicators: Age (years), UCd (μg/g cr), UNAG (U/g cr) and Uβ_2_-MG (μg/g cr). Age is expressed as the arithmetic mean (standard deviation): UCd, UNAG and Uβ_2_-MG are expressed as geometric mean (geometric standard deviation)

The geometric mean for the female UCd level (4.522 μg/g cr) was higher than that for the males (4.483 μg/g cr) (*p* < 0.05) (Table 2). Per clinical standards, patients are termed at risk if their UCd levels are higher than 5.000 μg/g cr. In our study, the median UCd value for both male or female participants was higher than the national standard threshold value (5 μg/g cr) recommended by GOMOHC in 2010 [31].

**Table 2:** Comparison of UCd concentrations and prevalence of UCd in population with different demographic characteristics

Characteristics	No.	Mean (GSD)	P_50_(P_25 ~ 75_)	P_95_	Abnormal number	X^2^	P
					[Percentage(%)]		
Gender						0.302	<0.05
Male	587	4.483(3.786)	5.751(2.463 ~ 8.077)	12.263	328(55.88)		
Female	1008	4.522(3.896)	5.700(3.542 ~ 8.156)	13.520	530(52.58)		
Age						747.000	<0.001
50~	589	4.401(3.657)	5.576(2.471 ~ 7.935)	12.105	302(51.27)		
60~	687	4.494(3.833)	5.775(3.544 ~ 8.230)	12.399	367(53.42)		
70~	257	4.671(4.127)	5.803(3.585 ~ 8.213)	14.937	143(55.64)		
80~	62	5.038(4.558)	6.009(2.412 ~ 9.830)	17.398	35(56.45)		

The units of the above indicators: Abnormal number (person), UCd (μg / g cr), GSD (Geometric Standard Deviation). 5 μg / g cr is the criterion for determining abnormality of UCd

Additionally, for high UCd concentration level, more than 5% of male (12.263 μg/g cr) was found lower than of female (13.520 μg/g cr). It was found that the UCd for 53.79% of all participants had high values, meaning that half of the sample was at risk of high Cd exposure. The mean UCd values for those aged 80–90 years old were 2.5 the national threshold standard. The likelihood of high UCd increased with age (*p* < 0.001) (Table 2). The mean UNAG (22.433 U/g cr) was far above the national threshold value of 17 U/g cr, as was the mean Uβ_2_-MG (2122.665 μg/g cr) in relation to the national threshold value of 1000 μg/g cr). The 75th to 95th percentiles for UNAG (20.661–33.271 U/g cr) and Uβ_2_-MG (1176.22–1834.13 μg/g cr) concentrations were likewise high.

### Relationship between urinary cadmium burden and renal dysfunction index

Index UNAG and Uβ_2_-MG were outlined. The percentage of high UNAG among female participants was 32.34%, and among males, it was 26.58% (Table 3). The percentages were higher for Uβ_2_-MG, with 41.77% for females and 36.63% for males. Thus, a trend of elevated values in UCd levels and the indexes is mentioned above. The rate of abnormal UNAG among males was 53.49%, and that of females was 61.44% (Table 3). The Uβ_2_-MG values ranged from 17.44 to 67.44% among makes and from 20.41 to 71.90% among. Uβ_2_-MG was more sensitive than UNAG (*p* < 0.05), supported previous results that Uβ_2_-MG might be a more sensitive biomarker than UNAG for Cd exposure risk [32]. Moreover, the odds of UNAG and Uβ_2_-MG increased linearly with UCd levels (*p* < 0.01). A further χ^2^ test of the linear trend verified that the indexes related to UCd levels (*p* < 0.01). An interesting finding was that the relationship of index response to renal dysfunction in females was higher than in males for the same UCd burden (Table 3).

**Table 3:** Prevalence of renal dysfunction at different UCd levels in male and female

UCd	Male					Female				
	No.	UNAG +/−	%	Uβ_2_-MG +/−	%	No.	UNAG +/−	%	Uβ_2_-MG +/−	%
(0 ~ 2]	86	5/81	5.81	15/71	17.44	147	12/135	8.16	30/117	20.41
(2 ~ 5]	173	34/139	19.65	45/128	26.01	331	68/263	20.54	93/238	28.10
(5 ~ 10]	242	71/171	29.34	97/145	40.08	377	152/225	40.32	188/189	49.87
(10~	86	46/40	53.49	58/28	67.44	153	94/59	61.44	110/43	71.90
Total	587	156/431	26.58	215/372	36.63	1008	326/682	32.34	421/587	41.77
*X*^*2*^		56.115		58.462			130.485		156.434	
p		0.000		0.000			0.000		0.000	
Linear-*X*^*2*^		52.520		53.353			128.313		154.094	
p		0.000		0.000			0.000		0.000	

The units of the above indicators: UCd (μg/g cr), UNAG (U/g cr), Uβ_2_-MG (μg/g cr). Chi-squared linear trend test, *p* < 0.01. 5 μg / g cr is the criterion for determining abnormality of UCd. 17 U/g cr is the criterion for determining abnormality of UNAG. 1000 μg / g cr is the criterion for determining abnormality of Uβ_2_-MG

A linear regression indicated a positive correlation between age and UCd (*p* < 0.05) (Table 4). The correlation coefficient of the Uβ_2_-MG level was higher than of UNAG (*p* < 0.05). Furthermore, the positive correlations between UCd and age, gender, UNAG, and Uβ_2_-MG were validated using Pearson correlation analysis (*p* < 0.05), but no significant correlation was found for village site (*p* > 0.05) (Table 4). A positive correlation between UNAG and Uβ_2_-MG was also confirmed (*p* < 0.01).

**Table 4:** Pearson’s correlation coefficients of factors affecting UCd and renal dysfunction

Index	Gender	Village	UNAG	Uβ_2_-MG	UCd
Age	0.101^b^	0.001	0.035	0.04	0.055^a^
Gender	–	−0.005	−0.003	− 0.027	0.076^a^
Village	–	–	0.027	0.044	0.033
UNAG	–	–	–	0.258^b^	0.437^b^
Uβ_2_-MG	–	–	–	–	0.492^b^

^a^: Correlation is significant at the 0.05 level (two-tailed); ^b^: Correlation is significant at the 0.01 level (two-tailed). UCd, UNAG and Uβ_2_-MG were log-transformed for correlation analysis

The renal indicators UNAG and Uβ_2_-MG were considered to be dependent variables, and the age, sex, region, and UCd levels (statistically analyzed after logarithmic transformation obeying the normal distribution) were considered to be independent variables. The variables that were entered into the regression (age, UCd, UNAG, and Uβ_2_-MG) were subjected to multiple linear regression analyses based on linear regression analysis. Certain factors, such as age, sex, and environmental exposure, may have resulted in a misleading assessment of the relationship between UCd burden and renal indexes. A step-by-step linear regression analysis, found β’ values for UNAG and Uβ_2_-MG of 0.459 and 0.493, respectively (Table 5). It was also found that UNAG and Uβ_2_-MG were sensitive biomarkers, and both may serve as primary indexes for BMD calculations.

**Table 5:** Results of multiple linear regression analysis of factors affecting UCd levels and renal dysfunction

Variable	β (95%CI)	S^−^_X_	β’	t	p
UNAG					
Age	0.021 (−0.028 ~ 0.071)	0.025	0.019	0.843	0.399
UCd	1.130 (1.023 ~ 1.238)	0.055	0.459	20.600	0.000
Uβ_2_-MG	0.001 (0.000 ~ 0.002)	0.000	0.052	2.099	0.036
Uβ_2_-MG					
Age	1.728 (−1.177 ~ 4.632)	1.481	0.027	1.167	0.243
UCd	55.778 (48.657 ~ 62.899)	3.630	0.493	15.364	0.000
UNAG	3.095 (0.203 ~ 5.986)	1.474	0.054	2.099	0.036
UCd					
Age	0.031 (2.437 ~ 5.399)	0.011	0.067	2.666	0.008

*β* partial regression coefficient; *S*^*−*^_*X*_ standard error; *β’* standardized partial regression coefficient; *t* t value of T-test; *p p* value

### BMD of UCd for UNAG and Uβ_2_-MG

The optimized logistic model was selected when fitting UNAG or Uβ_2_-MG. The BMD of UCd was presented and grouped according to gender (Table 6). The BMR was set at 10%, and UNAG was employed. BMD and BMDL for male UCd were nearly 3.876 U/g cr and 3.486 U/g cr, respectively. For the same parameter, the BMD and BMDL for female UCd were 3.236 U/g cr and 2.998 U/g cr, respectively. Given the same parameters for Uβ_2_-MG, the BMD and BMDL for male UCd were nearly 2.784 μg/g cr and 2.506 μg/g cr, respectively, and the BMD and BMDL of female UCd were about 2.416 μg/g cr and 2.236 μg/g cr, respectively (Table 6). This indicates that the BMDL of female UCd was lower than that of the male, implying that females were more vulnerable than males for the same UCd burden.

**Table 6:** The BMDLs results of the UCd for UNAG and Uβ_2_-MG in male and female

Gender	Model Name	Dependent variable	AIC	Chi-square	P-value	BMD	BMDL
Male	**Logistic**	UNAG	629.41	3.66	0.16	3.876	3.486
Female	**Logistic**	UNAG	1141.52	5.50	0.06	3.236	2.998
Male	**Logistic**	Uβ_2_-MG	716.38	0.02	0.99	2.784	2.506
Female	**Logistic**	Uβ_2_-MG	1251.34	1.06	0.59	2.416	2.236

The units: UNAG (U/g cr), Uβ_2_-MG (μg/g cr)

Based on the previous research, more study of the precise indexes was performed. In order to ensure the completeness and representativeness of the experimental results, all the dichotomous models for BMDs were applied, with BMR set as 15, 20, 25, and 30%. The national standard (5 μg/g cr) was produced by setting BMR to 20%. To protect people’s health from high levels of Cd exposure, additional restrictions to the sensitivity index and BMD should be considered.

## Discussion

Exposure to excess Cd may pose health risks [33, 34]. Clinically ascertaining threshold concentrations of UCd is greatly important and aids understanding of the health risks. Sensitivity indexes for the UCd burden have been a topic of wide concerned [15, 21]. In response to our questionnaire, more than 50% of respondents indicated their awareness of suffering high Cd exposure for a certain time. The results of a preliminary investigation of UCd levels and sensitivity biomarkers are discussed here.

In our study, UCd values for females varied from 0.030 μg/g cr to 20.279 μg/g cr, higher than those found in past research, which has suggested the UCd levels in females ranged from 0.49 to 0.69 μg/g cr, as deduced from individuals aged 50–79 in the US [35]. It has been speculated that age is a factor that determines UCd levels [36], and this is validated here. We found that UCd levels in the population increased with age (Table 1).

Additionally, UCd levels were affected by gender. The mean UCd among females differed statistically significantly from that of males (Table 2). The odd number of female UCd was 1.62 times higher than that of male (*p* < 0.05) (Table 3). This indicates that the females had accumulated more UCd, perhaps due to increased mineral retention and increased consumption of Cd-contaminating food during pregnancy [37]. Recent work has shown that lower iron content in females than in males may be due to their different physiologies, which may allow for greater absorption of Cd by the female gastrointestinal tract [38, 39]. The suggestion in this study that females may be more sensitive to Cd-induced nephrotoxicity than males supports an earlier study [40, 41].

Although mean levels of UCd and renal dysfunction were different for all age groups, an obvious dose–response relationship existed between UCd and the odds of renal dysfunction. This indicates that Cd exposure can cause irreversible damage to the kidney [21, 42]. For average UCd level, the odds of female were lower than that of males (Table 2); the reason might come from the sample sizes. Moreover, it has been found that endogenous hormones contribute to the UCd burden [43, 44]. The participants in our survey were all native residents, and most had been living in the study site more than 25 years. Long-term Cd exposure has been reported, and renal dysfunction has already been proven [45, 46]. Uβ_2_-MG and UNAG are in use as biomarkers for clinical diagnosis [15, 21] and animal experiments [47, 48]. In the later stages of Cd-induced renal injury, the tubular epithelial cells may shrink, fall off, and even disappear, and the tissue source of UNAG is lost [49, 50]. The reabsorption function of the kidney is impaired when UNAG was released, and the excretion of Uβ_2_-MG may be greater than that of UNAG [51, 52], which may be why Uβ_2_-MG produces higher values than UNAG in biological monitoring. In addition, the correlation coefficients for Uβ_2_-MG affecting UCd were higher than those for UNAG (Table 4). Traditionally, Uβ_2_-MG could be more sensitive as a renal health effect indicator than UNAG.

A comparison of the threshold concentration of UNAG and Uβ_2_-MG is essential for assessing the BMD of UCd using index total protein, UNAG, and Uβ_2_-MG in non-occupationally endangered people [21, 53], and the relationship between the UCd burden and renal indexes was found to be statistically significant (*p* < 0.01), as cross-checked by different models.

It has been suggested that the accumulation and excretion of UNAG and Uβ_2_-MG increases significantly with the increase of Cd burden in the organism, and irreversible kidney damage becomes more serious as Cd exposure grows [52, 53].

In this study, subjects of different genders were divided into four groups according to UCd levels (0 to 2, 2 to 5, 5 to 10, and > 10 μg/g cr). The results for BMDs showed that odd had a significant dose–effect trend with the selected effects endpoint. UNAG and Uβ_2_-MG were found to have good fit to the logistic model through BMD calculation.

The BMDLs of UCd for UNAG and Uβ_2_-MG in females were lower than those for males according to the BMDs results (Table 6), which also indicated that females were more sensitive to Cd exposure than males.

To protect human health from the high Cd burden, greater restrictions on sensitivity biomarkers and the BMD should be considered. BMD values of UCd for different renal indexes were lower than the standard threshold (5 μg/g cr) (Table 6). More than 20% of people might be at risk for Cd exposure where the UCd level was around the national standard threshold value (5 μg/g cr) for BMR was 20% (Table 7). Following our results, we recommend a value of 2.2 μg/g cr.

**Table 7:** The BMDLs of the UCd based on different BMRs in male and female

Gender	Model Name	Dependent variable	BMD (BMR = 15%)	BMD (BMR = 15%)	BMD (BMR = 20%)	BMDL (BMR = 20%)	BMD (BMR = 25%)	BMDL (BMR = 25%)	BMD (BMR = 30%)	BMDL (BMR = 30%)
Male	**Logistic**	UNAG	5.251	4.740	6.447	5.818	7.526	6.777	8.527	7.656
Female	**Logistic**	UNAG	4.138	3.858	5.131	4.793	6.037	5.641	6.885	6.427
Male	**Logistic**	Uβ_2_-MG	3.909	3.525	4.934	4.446	5.890	5.296	6.798	6.097
Female	**Logistic**	Uβ_2_-MG	3.423	3.174	4.350	4.034	5.222	4.838	6.058	5.604

The units: UNAG (U/g cr), Uβ_2_-MG (μg/g cr).

## Conclusions

This study found that most participants at a Cd-contaminated site had suffered severe damage to their kidney function, and more than 50% were in a high-risk group for Cd pollution. Their UCd levels were significantly lower than the threshold suggested by World Health Organization and GOMOHC. Uβ_2_-MG is recommended as the sensitivity index for renal dysfunction, with 2.2 μg/g cr as the threshold for clinical diagnosis. Our findings suggest that Uβ_2_-MG is the better biomarker for exposure to Cd. Our findings provide a tool for understanding Cd exposure on large spatial scales, which could help assess the health risks of Cd burden. This study also deduced threshold UCd levels, which will support improved clinical diagnosis of Cd exposure.

## Data Availability

The datasets used and/or analyzed during the current study are available on request from the corresponding author.

## Abbreviations

UCd: Urinary cadmium
UNAG: Urinary N-acetyl-β-D-glucosidase
Uβ_2_-MG: Urinary β_2_-microglobulin
BMD: Benchmark dose
BMR: Benchmark dose response
BMDL: Benchmark dose low
BMDLs: Lower confidence limits
NOAEL: No observed adverse effect level
AIC: Akaike information criterion
GOMOHC: Guidelines for the Diagnosis and Treatment of Heavy Metal Pollution of China in 2010
MOHC: Minister of Health of the People’s Republic of China
